# Decision aids for second-line palliative chemotherapy: a randomised phase II multicentre trial

**DOI:** 10.1186/s12911-017-0529-y

**Published:** 2017-08-31

**Authors:** Linda J. M. Oostendorp, Petronella B. Ottevanger, A. Rogier T. Donders, Agnes J. van de Wouw, Ivonne J. H. Schoenaker, Tineke J. Smilde, Winette T. A. van der Graaf, Peep F. M. Stalmeier

**Affiliations:** 1 Department for Health Evidence, Radboudumc, Nijmegen, the Netherlands; 2 Department of Medical Oncology, Radboudumc, Nijmegen, the Netherlands; 3 Department of Internal Medicine, VieCuri Medical Centre, Venlo, the Netherlands; 4 Department of Internal Medicine, Isala Clinics, Zwolle, the Netherlands; 5 Department of Medical Oncology, Jeroen Bosch Hospital, Den Bosch, the Netherlands

**Keywords:** Breast cancer, Colorectal cancer, Oncology, Decision aids, Palliative chemotherapy, Second-line

## Abstract

**Background:**

There is increasing recognition of the delicate balance between the modest benefits of palliative chemotherapy and the burden of treatment. Decision aids (DAs) can potentially help patients with advanced cancer with these difficult treatment decisions, but providing detailed information could have an adverse impact on patients' well-being. The objective of this randomised phase II study was to evaluate the safety and efficacy of DAs for patients with advanced cancer considering second-line chemotherapy.

**Methods:**

Patients with advanced breast or colorectal cancer considering second-line treatment were randomly assigned to usual care (control group) or usual care plus a DA (intervention group) in a 1:2 ratio. A nurse offered a DA with information on adverse events, tumour response and survival. Outcome measures included patient-reported well-being (primary outcome: anxiety) and quality of the decision-making process and the resulting choice.

**Results:**

Of 128 patients randomised, 45 were assigned to the control group and 83 to the intervention group. Median age was 62 years (range 32-81), 63% were female, and 73% had colorectal cancer. The large majority of patients preferred treatment with chemotherapy (87%) and subsequently commenced treatment with chemotherapy (86%). No adverse impact on patients' well-being was found and nurses reported that consultations in which the DAs were offered went well. Being offered the DA was associated with stronger treatment preferences (3.0 vs. 2.5; p=0.030) and increased subjective knowledge (6.7 vs. 6.3; p=0.022). Objective knowledge, risk perception and perceived involvement were comparable between the groups.

**Conclusions:**

DAs containing detailed risk information on second-line palliative treatment could be delivered to patients with advanced cancer without having an adverse impact on patient well-being. Surprisingly, the DAs only marginally improved the quality of the decision-making process. The effectiveness of DAs for palliative treatment decisions needs further exploration.

**Trial registration:**

Netherlands Trial Registry (NTR): NTR1113 (registered on 2 November 2007)

**Electronic supplementary material:**

The online version of this article (10.1186/s12911-017-0529-y) contains supplementary material, which is available to authorized users.

## Background

While patients with advanced cancer beyond cure are commonly offered palliative chemotherapy, there is increasing recognition of the delicate balance between the modest benefits of palliative chemotherapy and the burden of treatment [1–6]. Survival gains are usually in the range of weeks or months [7, 8], and while palliative chemotherapy can relieve symptoms and enhance quality of life [9], receiving palliative chemotherapy near the end of life has also been associated with receiving more aggressive medical care and worse quality of death [3, 10]. Furthermore, after failure of a first line of chemotherapy given with palliative intent, benefits of further lines of chemotherapy tend to be more limited. Therefore, starting first-line and particularly further-line palliative chemotherapy in addition to best supportive care should be carefully considered and any treatment implemented should be in harmony with the patient's preferences [5, 11, 12].

Alarmingly, in recent studies from the US, 52% and 69-81% of patients receiving palliative chemotherapy seemed to be under the false impression that this treatment may well cure their cancer [13, 14]. This inaccurate understanding may be related to incomplete communication by the oncologists, as well as patients' inability to accept the incurable nature of their disease [14]. Indeed, there are indications that clinicians and patients purposefully use a strategy of 'collusion' to communicate about the future, including avoiding or delaying the discussion of estimated life expectancy, in an effort to preserve patients' hope [15–19].

Decision aids (DAs) with information about risks and benefits of treatment options can potentially support patients in these difficult treatment choices. There is solid evidence from diverse healthcare settings that DAs can help patients to increase their knowledge and establish realistic expectations, and become more involved in making treatment decisions [20]. While designed to facilitate patient centered care, offering DAs with detailed information about estimated life expectancy for treatment with and without palliative chemotherapy may be at odds with clinicians' and patients' preferred strategy of 'collusion'. Studies have indicated that patients with a worse prognosis usually preferred less information and a less active role in decision-making [21, 22], while having prognostic discussions and encouraging patients to become more involved have been shown to be associated with increased anxiety among patients receiving or eligible for palliative chemotherapy [13, 23, 24]. Increased anxiety was also reported by patients who initially welcomed detailed prognostic information [13].

Several DAs have been developed to support decisions about palliative chemotherapy, most of them pertaining to decisions about first-line treatment [25–33]. Encouragingly, this series of mostly pilot studies have generally demonstrated good acceptability, although some patients thought the information was sad or too frank and caused distress or did not promote hope [25, 27, 28]. Beneficial effects included improved knowledge [28, 29, 31, 33] and stronger treatment preferences [30]. In addition, a number of DAs have been developed for advance care planning (i.e. future decisions) in patients with (advanced) cancer [34–37]. Acceptability of these DAs was generally high [35–37] and users demonstrated higher levels of knowledge [37] while their levels of hope and anxiety remained unchanged [35].

The aim of this study is to evaluate the feasibility of offering DAs containing detailed information about estimated life expectancy to patients with advanced breast or colorectal cancer considering second-line palliative chemotherapy, using a randomised multicentre design. Our primary aim was to evaluate any harmful effects of the DAs as compared with usual care, regarding patients’ well-being and specifically anxiety. Given patients' potential vulnerability, information was not routinely offered but patients were asked whether they wished to receive detailed information from the DA. In addition, we explored whether the previously reported beneficial effects of DAs also apply to palliative treatment decisions.

## Methods

### Study design

This randomised phase II study evaluating the feasibility of decision aids for second-line palliative chemotherapy was conducted in 17 hospitals in the Netherlands. The study was prospectively registered (Netherlands Trial Registry; NTR1113 http://www.trialregister.nl/trialreg/admin/rctview.asp?TC=1113), and a detailed account of the study design is available in the study protocol [38] (see Additional file 1). In summary, the decision aid offered information for patients considering second-line palliative chemotherapy for advanced breast or colorectal cancer. To identify patients who would be offered second-line palliative chemotherapy, we approached patients receiving first-line palliative chemotherapy for advanced incurable disease. Patients were excluded in case of a labile personality structure (as assessed by the medical oncologist), a Karnofsky performance score lower than 60, and insufficient Dutch language proficiency.

### Recruitment

Initial screening of potentially eligible patients against the selection criteria was performed by a medical oncologist or nurse. Eligible patients were approached by the health professional to ask permission for a researcher to contact them about a study testing a new way of providing information to patients. Importantly, health professionals did not mention that explicit information on expected survival would be offered to patients, in an effort to prevent selection of patients not wishing to receive such information.

### Procedure

When a patient included in the study experienced disease progression and was offered second-line chemotherapy, randomisation was performed. A nurse would open a sealed envelope to find out whether the patient would either: (1) be informed by the oncologist in the usual way (control group); or (2) be informed by the oncologist in the usual way followed by a consultation with a nurse offering a DA (intervention group). Unequal randomisation (using a 1:2 ratio) was used because the sample size of the control group was based on the current evaluation of the DAs, while the sample size of the intervention group was based on more detailed analyses of patients' desire for information [38, 39]. Randomisation lists were computer generated per hospital and tumour type, using a block size of 3. Patients in the intervention group were offered an appointment with a nurse to receive the DA, typically within a week after the oncologist imparted the news of disease progression and discussed treatment options (depending on local workflow and patient preferences).

### The DAs

**Fig. 1:**
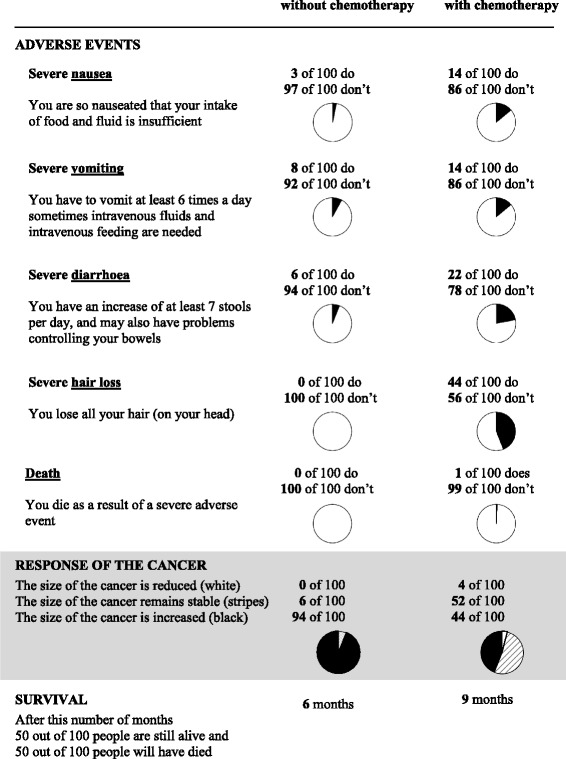
Example of a summary page of a DA for colorectal cancer

The DA booklets were designed based on our previous experience with DAs for prostate cancer treatment [40, 41], and we followed guidance from the International Patient Decision Aids Standards where possible [42]. The booklets started with an introduction describing both treatment options and showing an example of numerical information provided. In the next section, numerical information was provided on: (1) the incidence of adverse events; (2) the chances of achieving a tumour response; and (3) expected median survival. This information was derived from systematic reviews of the literature for the two tumour types [7, 8], and tailored to the particular type of chemotherapy offered to the patient (for a total of eleven types of chemotherapy). Figure 1 shows an example of the information shown in a DA for second-line irinotecan; a f﻿ull copy of a DA is available in Additional file 2.

### Measures

Oncologists completed an inclusion form (see Additional file 3) and nurses completed a brief questionnaire about the interview with the DA (see Additional file 4). Patients were asked to complete a first questionnaire at inclusion (T1) and patients in both groups were asked to complete two follow-up questionnaires one week (T2) and eight weeks (T3) after receiving treatment-related information. Patient questionnaires are available in Additional files 5, 6 and 7.

#### Sociodemographic variables and medical history

Patient’s gender and tumour type were extracted from the inclusion form. The baseline questionnaire contained questions on age, level of education, marital and working status, and having children or grandchildren.

#### Intervention interview

Nurses were given a paper-and-pencil questionnaire divided into two sections (see Additional file 4). The first section was completed during the interview with the patient and started with a question about treatment preference and strength of this preference. After that, the nurse administered the DA.

The nurse first introduced the DA and the two options for second-line treatment (best supportive care with or without second-line palliative chemotherapy) and showed an example of risk information. The nurse then proceeded to offer information for the first item, adverse events. First the patient was given a brief explanation of the type of information to be expected (e.g. implications of a severe adverse event) and then the patient could indicate whether he or she wished to receive the information. The information was provided accordingly. A booklet with information tailored to the patient’s desire was available to take home.

To conclude the first section of the interview, the nurse asked the patient once more about treatment preference and strength of this preference. Nurses were instructed to complete the second section after the consultation with the patient. This section included a question about how the interview went, and the nurse was asked to record which chemotherapeutic drug was discussed with the patient.

#### Outcome measures

Since this is one of the first randomised studies evaluating the safety and efficacy of a DA in a population of advanced cancer patients deciding about palliative chemotherapy, we decided to explore a broad range of outcomes to assess safety in this potentially vulnerable population, and assess whether the previously found beneficial effects can be replicated in this population. Therefore the selection of outcome measures was largely based on outcomes used in previous research [43].

Given the concerns about anxiety in this potentially vulnerable patient population, the primary outcome of this study was selected to be anxiety. At the time the sample size calculation was conducted, no information was available about the minimal clinically important difference for the HADS anxiety scale. We had to resort to an educated guess and decided to use a difference of 2.2, equivalent to a difference of >10% on a scale of 0-21. Recent studies, albeit in populations of patients with COPD and survivors of acute respiratory failure [44–46], seem to indicate that the estimate of 2.2 was not unreasonable. The primary outcome measure anxiety was defined a priori, and all main and additional outcome measures have been described beforehand in the publicly available study protocol [38]. The outcomes of our exploratory analysis of additional outcome measures were interpreted with appropriate caution, and any statistically significant findings will need to be confirmed by future studies [47]. An overview of outcome measures and operationalisation is shown in Table 1.

**Table 1:** Overview of outcome measures

		Timing of measurements^1^		
Measures	Operationalisation	Baseline T1	1 week follow-up T2	8 weeks follow-up T3
Main outcome measures				
Well-being				
Anxiety^2^	Hospital Anxiety and Depression Scale	x	x	x
Depression	Hospital Anxiety and Depression Scale	x	x	x
General health	0-10 (worst-best imaginable)	x	x	x
Cancer Worries	Adapted Lerman’s Cancer Worry Scale	x	x	x
Health-related quality of life	EORTC QLQ-C15-PAL	x	x	x
Additional outcome measures				
Coping				
Helplessness/hopelessness	Mental Adjustment to Cancer Scale	x	x	x
Fighting spirit	Mental Adjustment to Cancer Scale	x	x	x
Avoidance	Mental Adjustment to Cancer Scale	x	x	x
Perceived participation	Problem-Solving Decision Making Scale		x	x
Perceived involvement	yes/no		x	x
Information-related measures				
Amount of information	1-7 (I received way too little-way too much information)	x	x	x
Undesired information	yes/no		x	
Satisfaction with quality of information	1-6 (not satisfied-very much satisfied)		x	x
Balanced presentation of information	1-5 (clearly in favour of chemotherapy plus BSC - clearly in favour of BSC alone)		x	
Evaluation of information	1-5 (no negative experience-very negative experience)		x	
Knowledge				
Subjective knowledge	1-10 (extremely poor-excellent)	x	x	
Objective knowledge	five statements to be judged as right or wrong		x	
Subjective risk perception	1-5 (very high-very low)^3^		x	
	1-7 (much higher-much lower)^4^		x	
Objective risk perception	0-100%^5^		x	
Decision-related measures				
Decision satisfaction-uncertainty	Decision Evaluation Scales		x	x
Decision control	Decision Evaluation Scales		x	x
Weighing pros and cons	Decision Evaluation Scales		x	x
Treatment choice	chemotherapy + BSC /BSC alone/don’t know		x	x
Strength of treatment preference	1-5 (not strong-very strong)^6^		x	
Treatment attitudes				
Valuations	1-10 (extremely poor-excellent)		x	x
Treatment satisfaction	1-6 (dissatisfied-very satisfied)			x

*Abbreviation*: *BSC* best supportive care

^1^Baseline: at inclusion; follow-up: 1 and 8 weeks after receiving the treatment-related information

^2^Anxiety is the primary outcome measure

^3^Question 1: 'the chance of experiencing an adverse event'

^4^Question 2: 'the chance of experiencing a beneficial effect on the tumour when having treatment with chemotherapy and BSC, as compared with BSC alone' and question 3 'the chance of experiencing pain when having treatment with chemotherapy and BSC, as compared with BSC alone'

^5^The absolute deviation between patient's objective risk perception and the actual risk (as identified in the literature reviews [7, 8] and presented in the DAs) was calculated.

^6^For patients whose treatment choice was ‘undecided’, the strength of the treatment preference was scored as zero

##### Main outcome measures

The primary outcome measure of this study was anxiety [48]. Four other measures of patient well-being were used, including general health, health-related quality of life (HRQoL) [49], depression [48], and cancer worries [50].

##### Additional outcome measures

*Coping*

Patients were asked questions on their mental adjustment to cancer, including their style of coping including helplessness/hopelessness, fighting spirit, and avoidance [51]. In addition, patients were asked questions on perceived participation and perceived involvement including the perception of being offered a choice and the perception that their opinion mattered [52, 53].

*Information-related measures*

Questions were asked about the amount of treatment-related information received and about receiving any undesired information. Furthermore, patients were asked to rate the quality of information, whether treatment options were presented in a balanced way, and whether they had any negative experiences with the information received.

*Knowledge*

Patients rated their knowledge about cancer and its treatment, and were presented with five statements, judged to be right or wrong, to assess objective knowledge. For subjective risk perception, patients were asked to rate the following chances: (1) the chance of experiencing an adverse event, (2) the chance of experiencing a beneficial effect on the tumour when having treatment with chemotherapy and BSC, as compared with BSC alone, and (3) the chance of suffering from pain when opting for chemotherapy, as compared with BSC alone. Objective risk perception was assessed by asking for the chances of: (1) experiencing severe diarrhoea and (2) achieving partial or complete tumour response. The format of these questions was mostly based on knowledge questions in other studies [20] and our own previous work [40, 41] and the content was informed by key information provided in the decision aid, e.g. adverse events and tumour response.

*Decision-related measures*

The decision was evaluated with questions about patients' satisfaction and uncertainty around the decision, patients' sense of control in the treatment decision, and deliberation of pros and cons of treatment options [54]. Furthermore, patients were asked about their preferred treatment, and, if applicable, the strength of that preference (T2), and about the treatment they actually received (T3).

*Treatment attitudes*

Patients were asked about treatment attitudes because these are considered to be one of the main determinants of (health) behaviour, according to theories for behaviour and behaviour change [55]. Patients were asked to value each of the two treatment options on a scale of 1-10 (T2 and T3), and rate their satisfaction with (1) the implemented treatment; (2) the physical consequences of treatment and (3) the emotional consequences of treatment (1 'dissatisfied' - 6 'very satisfied') at T3.

### Statistical Analysis

To assess the risk of attrition bias, characteristics of randomised patients were compared with inadvertently non-randomised patients, using independent samples t-tests or Chi-Square tests, as applicable. The safety and efficacy of the DAs were assessed by comparing patients in the intervention and control groups, on an intention-to-treat basis. Nominal variables were analysed using the Chi-Square test of independence. Interval variables that were available for a single follow-up measurement were analysed using an independent samples t-test, Chi-Square test of independence or Fisher Exact Test or, if a baseline measurement was available, analysis of covariance (ANCOVA). Interval variables that were available for both follow-up measurements were analysed using linear mixed models. In these models, dependent variables were the two follow-up measurements (T2 and T3), and covariates were the variables ‘group’, ‘time’, an interaction term between 'group' and 'time' and if available, 'baseline measurement'. The covariates 'group*time', 'time', and 'baseline measurement' were stepwise removed from the model based on statistical significance. To accommodate the repeated measures we used a heterogeneous compound symmetry error structure.

## Results

### Patients

**Fig. 2:**
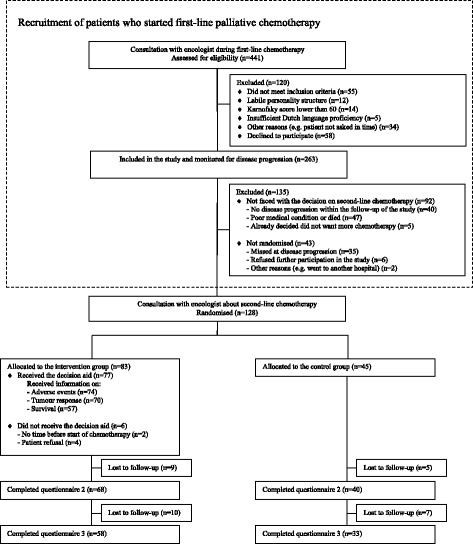
Flow diagram

Screening of potentially eligible patients took place between February 2008 and April 2012. As shown in Fig. 2, out of 441 patients screened, 34 patients (8%) were not approached by the oncologist and therefore the selection criteria could not be verified. Another 86 patients (20%) did not fulfil the selection criteria. Out of the remaining 321 patients, 263 (82%) agreed to participate and gave informed consent.

Over time, 171 included patients (65%) faced the decision on second-line treatment and were eligible for randomisation. However, 43 of them (16%) were not randomised and dropped out of the study. To assess selective attrition, we compared characteristics between these 43 patients and the 128 patients who were randomised and found that patients were similar with regard to gender, age, education, tumour type, information preference and general health.

Out of 128 patients randomised, 45 were randomly assigned to the control group and 83 were assigned to receiving the DA in addition to usual care. Characteristics of randomised patients are shown in Table 2. The two groups were similar with regard to baseline characteristics.

**Table 2:** Patient characteristics

	Intervention group (n=83)	Control group (n=45)
Male gender, n (%)	31 (37%)	16 (36%)
Age, mean (SD)	61 (9.1)	62.5 (9.5)
Living with partner, n (%)	64 (77%)	37 (82%)
Employed, n (%)	25 (30%)	13 (29%)
Having children, n (%)	74 (89%)	38 (84%)
Having grandchildren, n (%)	44 (53%)	26 (58%)
College education or more, n (%)	24 (29%)	7 (16%)
Tumour characteristics		
*Colorectal cancer, n (%)*	61 (73%)	32 (71%)
*Breast cancer, n (%)*	22 (27%)	13 (29%)

### Intervention interview

In the intervention group, 77 patients (93%) completed the intervention interview with a nurse offering the DA. A total of 20 nurses (between 1 and 3 per hospital) were involved in conducting the intervention interviews (median number of interviews conducted: 2; range 1-22). The majority opted to be shown the information in the DA with regard to (1) adverse events (96%); (2) tumour response (91%); and (3) survival (74%). Nurses felt that 90% of the interviews went (very) well.

### Outcomes

Table 3 provides a comparison of outcomes for the main outcome measures and additional outcomes measured at both follow-up moments (T2 and T3), analysed using linear mixed models. The variable ‘group’ was retained in all models to assess the effect of the DA. Table 4 provides a comparison of additional outcomes between groups at a single follow-up moment (T2 or T3).

**Table 3:** Comparison of outcomes over multiple time points (T2 and T3)

				Linear mixed models		
Measure (answer scale)^1^		Intervention group^2^ Mean (SD)	Control group^2^ Mean (SD)	Covariates in model	Difference between intervention and control group averaged over T2 and T3, adjusted for T1^3^ (95% CI)	Two- sided p value
Main outcome measures						
Well-being						
Anxiety^4^ (0-21 )	T1	5.7 (4.0)	5.6 (4.0)	Group	-0.1 (-1.1;0.9)	*0.808*
	T2	6.6 (4.5)	6.1 (4.5)	Baseline		
	T3	5.5 (4.1)	5.9 (4.6)	Time		
Depression (0-21)	T1	5.1 (3.6)	4.1 (3.0)	Group	-0.7 (-1.7;0.3)	*0.142*
	T2	5.6 (4.2)	5.6 (4.2)	Baseline		
	T3	5.3 (3.6)	5.2 (3.5)			
General health (0-10)	T1	6.5 (1.6)	7.1 (1.7)	Group	-0.2 (-0.8;0.5)	*0.615*
	T2	5.7 (1.8)	6.1 (1.9)	Baseline		
	T3	5.8 (2.0)	5.9 (1.7)			
Cancer worries (1-4)	T1	2.1 (0.6)	2.0 (0.6)	Group	-0.1 (-0.3;0.1)	*0.192*
	T2	2.1 (0.5)	2.1 (0.6)	Baseline		
	T3	2.1 (0.6)	2.2 (0.7)			
HRQoL: physical functioning (0-100)	T1	75.2 (22.3)	79.2 (18.1)	Group	-2.2 (-9.2;4.8)	*0.536*
	T2	68.2 (22.5)	72.3 (21.0)	Baseline		
	T3	67.9 (25.0)	71.1 (22.1)			
HRQoL: emotional functioning (0-100)	T1	81.0 (22.0)	80.5 (20.2)	Group	3.5 (-3.4;10.5)	*0.318*
	T2	74.3 (22.0)	73.6 (27.1)	Baseline		
	T3	79.7 (20.2)	75.5 (25.2)			
Additional outcome measures						
Coping						
Helplessness/Hopelessness (1-4)	T1	1.8 (0.6)	1.7 (0.5)	Group	0.0 (-0.1;0.2)	*0.759*
	T2	1.8 (0.6)	1.8 (0.5)	Baseline		
	T3	1.9 (0.6)	1.8 (0.5)			
Fighting Spirit (1-4)	T1	2.9 (0.6)	3.2 (0.6)	Group	-0.0 (-0.2;0.1)	*0.568*
	T2	2.7 (0.6)	3.0 (0.6)	Baseline		
	T3	2.8 (0.7)	2.8 (0.7)			
Avoidance (1-4)	T1	2.5 (0.7)	2.6 (0.8)	Group	0.1 (-0.0;0.3)	*0.094*
	T2	2.5 (0.7)	2.6 (0.7)	Baseline		
	T3	2.5 (0.7)	2.3 (0.7)			
Perceived participation (1-5)	T2	3.1 (1.0)	2.8 (0.9)	Group	0.2 (-0.2;0.5)	*0.395*
	T3	2.9 (1.0)	2.9 (0.8)			
Information-related measures						
Amount of information received (1-7)	T1	3.8 (0.6)	3.9 (0.3)	Group	-0.1 (-0.3;0.0)	*0.157*
	T2	3.8 (0.7)	4.0 (0.4)	Baseline		
	T3	3.8 (0.5)	3.9 (0.3)			
Satisfaction with quality of information (1-6)						
Severe adverse events	T2	4.8 (0.9)	4.8 (1.0)	Group	0.0 (-0.3;0.4)	*0.802*
	T3	4.5 (1.2)	4.4 (1.1)	Time		
Tumour response	T2	4.5 (1.0)	4.4 (1.2)	Group	0.1 (-0.2;0.5)	*0.536*
	T3	4.3 (1.1)	4.2 (1.1)			
Survival	T2	4.0 (1.3)	4.0 (1.3)	Group	0.1 (-0.3;0.6)	*0.540*
	T3	4.0 (1.2)	3.6 (1.4)			
Knowledge						
Subjective knowledge^5^	T1	6.5 (1.2)	6.6 (1.0)	Group	0.5 (0.1; 0.9)	*0.022*
	T2	6.7 (1.2)	6.3 (1.3)	Baseline		
Decision-related measures						
Decision satisfaction-uncertainty (1-5 )	T2	4.1 (0.6)	4.0 (0.7)	Group	0.1 (-0.1;0.3)	*0.155*
	T3	4.1 (0.6)	3.9 (0.4)			
Decision control (1-5)	T2	4.2 (0.7)	4.3 (0.6)	Group	-0.1 (-0.3;0.2)	*0.617*
	T3	4.3 (0.6)	4.3 (0.6)			
Weighing pros and cons (1-5)	T2	4.2 (0.8)	3.9 (1.0)	Group	0.2 (-0.1;0.5)	*0.118*
	T3	4.0 (1.1)	3.8 (0.8)			
Treatment attitudes toward both options						
Valuations: chemotherapy + BSC (1-10)	T2	7.5 (1.7)	7.2 (1.6)	Group	0.1 (-0.5;0.7)	*0.677*
	T3	7.1 (1.8)	7.3 (1.7)			
Valuations: BSC alone (1-10)	T2	3.7 (2.1)	4.3 (2.1)	Group	-0.4 (-1.2;0.3)	*0.246*
	T3	4.1 (2.3)	4.6 (2.4)			

*Abbreviations*: *SD* standard deviation, *CI* confidence interval, *BSC* best supportive care

^1^More information on the scales can be found in Table 1

^2^Intervention group: T1 n=82, T2 n=68, T3 n=58 Control group: T1 n=44, T2 n=40, T3 n=33

^3^Positive numbers represent higher scores in the intervention group

^4^Anxiety is the primary outcome measure

^5^Analysed using analysis of covariance (ANCOVA); difference between intervention and control group at T2, adjusted for T1

**Table 4:** Comparison of outcomes at a single time point (T2 or T3)

Measure (answer scale)^1^	Intervention group N (%) or mean (SD)	Control group N (%) or mean (SD)	*p* value
A dditional outcome measures: measurements at T2	n=68	n=40	
Coping: Involvement			
Perceived involvement: perception of being offered a choice (yes/no)^2^	45 (66%)	26 (67%)	*0.959*
Perceived involvement: perception whether patient’s opinion mattered (yes/no)^2^	51 (75%)	30 (77%)	*0.823*
Information-related measures			
Undesired information (yes/no)	6 (10%)	7 (18%)	*0.244*
Balanced presentation of information (1-5)	2.7 (0.7)	2.4 (1.1)	*0.201*
Evaluation of information on treatment options: unpleasant (1-5)	2.1 (1.0)	2.2 (1.2)	*0.679*
Evaluation of information on treatment options: shocking (1-5)	2.3 (1.0)	2.3 (1.0)	*0.958*
Evaluation of information on treatment options: frightening (1-5)	2.4 (0.9)	2.2 (1.0)	*0.347*
Evaluation of information on severe adverse events: threatening (1-5)	2.5 (1.0)	2.7 (1.1)	*0.358*
Evaluation of information on tumour response: threatening (1-5)	2.7 (1.1)	2.7 (1.0)	*0.786*
Evaluation of information on survival: threatening (1-5)	3.0 (1.3)	2.6 (1.3)	*0.112*
Knowledge			
Objective knowledge (1-5)	3.4 (1.3)	3.5 (1.3)	*0.684*
Subjective risk perception: severe adverse events (1-5)	2.2 (0.7)	2.0 (0.8)	*0.242*
Subjective risk perception: tumour response (1-7)	1.8 (1.1)	2.1 (1.1)	*0.219*
Subjective risk perception: pain (1-7)	3.0 (1.2)	3.2 (1.3)	*0.461*
Objective risk perception: diarrhoea (0-100%)^3^	30.9 (22.1)	34.9 (22.1)	*0.366*
Objective risk perception: tumour response (0-100%)^3^	30.0 (20.8)	32.5 (14.3)	*0.463*
Decision-related measures			
Treatment choice: undecided (vs. decided)	1 (2%)	4 (10%)	*0.068*
Treatment choice: chemotherapy (vs. no chemotherapy)	63 (96%)	31 (84%)	*0.067*
Strength of treatment preference (1-5)	3.0 (1.0)	2.5 (1.2)	*0.030*
Additional outcome measures: measurements at T3	n=58	n=33	
Coping: Involvement			
Perceived involvement: perception of being offered a choice (yes/no)^2^	41 (71%)	20 (61%)	*0.299*
Perceived involvement: perception whether patient’s opinion mattered (yes/no)^2^	47 (81%)	25 (76%)	*0.525*
Decision-related measures			
Treatment received: chemotherapy and BSC (vs. BSC alone)	50 (88%)	26 (84%)	*0.746*
Treatment attitudes toward the treatment received			
Treatment satisfaction: received treatment (1-6)	4.6 (1.3)	4.6 (1.0)	*0.794*
Treatment satisfaction: physical consequences of treatment (1-6)	3.8 (1.5)	3.5 (1.3)	*0.372*
Treatment satisfaction: emotional consequences of treatment (1-6)	4.0 (1.4)	3.9 (1.3)	*0.725*

*Abbreviation*: *BSC* best supportive care

^1^More information on the scales can be found in Table 1

^2^Measured at T2 and T3

^3^Objective risk perception represents the absolute deviance between patient’s risk perception and actual risk as presented in the DA, based on literature reviews [7, 8]

#### Main outcome measures

Receiving the DA was not related to any of the measures for well-being, including the primary outcome anxiety (Table 3). In both groups, average anxiety scores were approximately 6 on a 0-21 scale, and the difference between the groups was -0.1 (95% CI -1.1;0.9). When using a threshold of 8 or 11 [56], heightened levels of anxiety and/or depression were detected in 6-33% of all patients at T1, 10-38% of patients at T2 and 10-33% of patients at T3, which is similar to other populations of patients with advanced cancer.

#### Additional outcome measures

##### Coping

Patients in the intervention and control groups reported equal levels of helplessness/hopelessness, fighting spirit, avoidance, and perceived participation (Table 3) and perceived involvement (Table 4).

##### Information-related measures

No differences were found between the groups over time for the amount of information and satisfaction with the quality of information (Table 3). Patients in both groups responded similarly to questions about undesired information, balanced presentation, and evaluation of the information (Table 4).

##### Knowledge

Patients in the intervention group felt significantly more knowledgeable compared with patients in the control group (6.7 vs. 6.3; p=0.022). Objective knowledge and subjective and objective risk perceptions were similar between the groups (Table 4).

##### Decision-related measures

At the first follow-up (T2) the large majority of patients reported having a treatment preference (95%); most of them favoured chemotherapy (87%). The two groups responded similarly to questions about decision satisfaction and uncertainty, decision control, and weighing of pros and cons (Table 3). Patients in the intervention group reported stronger treatment preferences (3.0 vs. 2.5; p=0.030). At T3, there were no differences in treatment received between the groups (Table 4).

##### Treatment attitudes

Valuations of both treatment options were comparable between the groups (Table 3). In both groups, treatment with chemotherapy was on average valued with a 7.4 while treatment with BSC alone was valued with a 4.0 (on a scale of 1-10), resulting in a mean difference of 3.4 (95% CI 2.8-4.1; p=0.000*)*. As shown in Table 4, patients in both groups felt equally satisfied with their treatment and the consequences of treatment (both physical and emotional).

## Discussion

This study was designed to address concerns about offering detailed treatment-related information to a potentially vulnerable group of patients with advanced cancer [13, 18, 19, 23]. In line with previous research in a wide variety of patient populations [20] and patients with advanced cancer in an earlier stage of the treatment trajectory [25, 33, 57], this study has not revealed an adverse impact of decision aids in this patient population. The large majority of patients opted to be shown all of the available detailed information in the decision aid and nurses reported that nearly all consultations went well. Follow-up at 1 and 8 weeks did not reveal any harmful effects on anxiety or other measures of well-being, including cancer worries, nor was receiving the decision aid associated with having received undesired information, reporting a more negative experience (e.g. receiving threatening or frightening information), or increased feelings of hopelessness.

With regard to efficacy, patients who were offered a DA were found to have better subjective knowledge and stronger treatment preferences. These are important benefits. However, previous studies had also reported beneficial effects regarding objective knowledge, risk perception, and involvement in decision-making [40, 41, 58–60] and these could not be confirmed. The question arises why these benefits could not be confirmed in our study. Several explanations are possible, and might be related to: (1) the population of patients with advanced cancer considering second-line palliative chemotherapy; (2) the decision aids; or (3) the design of our trial.

First, in our interpretation, a similar relative absence of effects was reported in the single randomised study evaluating a DA on first-line palliative chemotherapy; a positive effect was reported for objective knowledge, but no positive effects were found for other commonly used measures such as involvement [33]. Therefore, we might contribute the absence of effects to the particular characteristics of the population of vulnerable patients with advanced cancer. Perhaps these patients did not perceive the option to refrain from active treatment as a realistic option. This has been suggested before [5, 61–64]. Also data from our trial seem to suggest so, as patients valued the appropriateness of best supportive care alone significantly lower than best supportive care with chemotherapy. Furthermore, a number of patients wrote down open-ended comments on the patient questionnaires, including 'I cannot value the appropriateness of BSC, because I have not discussed it or thought about it', 'I do not have a choice', and 'The only thing that matters is the effect of chemotherapy, adverse events do not matter to me'. Previous research has shown that particularly for second-line treatment, an important reason to opt for active treatment is to promote hope [62–64].

Second, the DAs were offered by 20 different nurses and after the consultation with the oncologist; timing was tailored to local workflows and DAs may have been offered relatively late in the decision-making process, which may have reduced their value. It could also be hypothesized that the added value of the DAs was reduced because all patients in this study had previous experience with chemotherapy. Third, another explanation could be related to a lack of statistical power of this study since power was reduced by patient attrition at T2 and T3, however none of the differences between the groups appears large enough to be clinically relevant.

One of the strengths of this study is that we performed one of the few randomised evaluations of a DA for palliative chemotherapy, and the first exploratory evaluation for second-line treatment options. A further strength is that we facilitated generalisability of the results by recruiting patients from a large number of hospitals, recruiting patients receiving first-line chemotherapy while applying broad selection criteria, and achieving an 82% informed consent rate. Thorough attention was paid to including evidence-based information in the DAs, by performing systematic reviews which were subjected to peer-review [7, 8].

Noteworthy limitations include multiple testing. A total of 52 comparisons between the intervention and control group were performed, of which two (4%) reached statistical significance at the level p<0.05. These two findings need to be interpreted with caution. Another limitation inherent to the nature of DAs is that complete blinding was not possible. Nevertheless, oncologists were not aware of the allocation prior to randomisation and our analysis showed no differences in clinical and sociodemographic characteristics between randomised and non-randomised patients.

Future studies might explore patients' perceptions of palliative treatment choices, including ways to make patients aware that best supportive care without chemotherapy is a realistic treatment option. Studies might aim to close the decision support loop by scheduling a consultation with the oncologist after the DA is offered. This allows patients time to think and have an informed discussion with their oncologist. With regard to the presentation of survival information, developers of future DAs should consider offering patients survival information using typical, best-case and worst-case scenarios instead of median survival [65].

## Conclusions

Decision aids with detailed information on risks and benefits of second-line palliative chemotherapy were welcomed by the majority of patients with advanced cancer and no adverse impact on patient well-being was observed. These decision support tools can help to provide standardised information about potential risks and benefits of available treatment options. While patients reported some beneficial effects, other previously reported benefits -including improvements in objective knowledge, risk perception and involvement in decision-making- were not confirmed for the decision aids in this study. Results from this explorative trial have indicated that even in a potentially vulnerable population of patients with advanced cancer beyond cure, decision aids with detailed treatment-related information can be offered, and this encouraging finding will hopefully stimulate further research in this field.

## Additional files

Additional file 1: Changes from published study protocol. Overview of changes in study design compared with the published protocol (PDF 640 kb)

Additional file 2: Decision aid for second-line irinotecan. Example of information provided in a decision aid. (PDF 89 kb)

Additional file 3: Inclusion and progression form. Copy of questionnaire completed by clinicians at inclusion and progression. (PDF 79 kb)

Additional file 4: Questionnaire decision aid. Copy of nurse questionnaire. (PDF 89 kb)

Additional file 5: Patient questionnaire 1. Copy of patient questionnaire 1 (T1, baseline). (PDF 236 kb)

Additional file 6: Patient questionnaire 2. Copy of patient questionnaire 2 (T2, one week follow-up). (PDF 402 kb)

Additional file 7: Patient questionnaire 3. Copy of patient questionnaire 3 (T3, eight week follow-up). (PDF 103 kb)

## Abbreviations

ANCOVA: Analysis of covariance
BSC: Best supportive care
CI: Confidence interval
DA: Decision aid
NTR: Netherlands Trial Registry
SD: Standard deviation
