# Supplementary material for: Decision aids for second-line palliative chemotherapy: a randomised phase II multicentre trial
Source: BMC Med Inform Decis Mak. 2017 Aug 31;17:130. doi: 10.1186/s12911-017-0529-y (PMC5580234; doi:10.1186/s12911-017-0529-y)
Supplement: Supplementary file 1 — Changes from published study protocol. Overview of changes in study design compared with the published protocol (PDF 640 kb) [file 12911_2017_529_MOESM1_ESM.pdf]

Additional File 1 - Changes from published study protocol

The study protocol has been published in BMC Medical Informatics and Decision Making:

> Assessing the information desire of patients with advanced cancer by providing information with a decision aid, which is evaluated in a randomized trial: a study protocol.
> Oostendorp LJ[1], Ottevanger PB, van der Graaf WT, Stalmeier PF.
> BMC Med Inform Decis Mak. 2011 Feb 14;11:9. doi: 10.1186/1472-6947-11-9.

Overview of important changes to methods after trial commencement

1. Number of recruiting sites

   In the published study protocol we described how patients would be recruited from 11 hospitals. We decided to increase the number of hospitals to 17, mainly because patients could not be randomised until they experienced disease progression and for several patients this interval was longer than expected. As shown in the flow diagram in Figure 2, 40 patients did not experience disease progression within the follow-up of the study.

2. Ovarian cancer

   We set out to develop decision aids for patients with advanced colorectal, breast and ovarian cancer. Development of the decision aids took longer than expected and included conducting and publishing two comprehensive systematic reviews of the literature:

   Systematic review of benefits and risks of second-line irinotecan monotherapy for advanced colorectal cancer.
   Oostendorp LJ, Stalmeier PF, Pasker-de Jong PC, Van der Graaf WT, Ottevanger PB.
   Anticancer Drugs. 2010 Sep;21(8):749-58. doi: 10.1097/CAD.0b013e32833c57cf. Review.

   Efficacy and safety of palliative chemotherapy for patients with advanced breast cancer pretreated with anthracyclines and taxanes: a systematic review.
   Oostendorp LJ, Stalmeier PF, Donders AR, van der Graaf WT, Ottevanger PB.
   Lancet Oncol. 2011 Oct;12(11):1053-61. doi: 10.1016/S1470-2045(11)70045-6. Epub 2011 May 27. Review.

   To meet timelines we decided to develop decision aids for colorectal cancer and breast cancer only. Including these two cancer types would yield the highest number of potentially eligible patients.

3. Sample size calculation

   Our initial sample size calculation was included in the published study protocol and stated that "70 patients were needed per group to identify a difference of 2.2 on the HADS anxiety scale (range 0-21) (power of 81%; two-sided α=0.05)". However, this calculation did not take the unequal randomisation ratio (1:2) into account. A post hoc sample size calculation showed that in order to obtain 80% power for a randomisation ratio of 1:2, a difference between the conditions of 2.2 points on the HADS anxiety scale with a standard deviation of 4.2 points, and a two sided testing procedure using an alpha of 0.05, 44 subjects needed to be randomised to the control condition and 88 patients needed to be randomised to the experimental condition.
