# Supplementary material for: Decision aids for second-line palliative chemotherapy: a randomised phase II multicentre trial
Source: BMC Med Inform Decis Mak. 2017 Aug 31;17:130. doi: 10.1186/s12911-017-0529-y (PMC5580234; doi:10.1186/s12911-017-0529-y)
Supplement: Supplementary file 2 — Decision aid for second-line irinotecan. Example of information provided in a decision aid. (PDF 89 kb) [file 12911_2017_529_MOESM2_ESM.pdf]

Decision aid

Copy for nurse

Information for patients with advanced colorectal cancer on the decision about two treatment options

UMC St Radboud

DUTCH CANCER SOCIETY

Introduction

Explaining the goal of the study

You are participating in a study on ways to inform patients like you about their treatment options. As a part of this study, we have collected information on the available treatment options and developed this decision aid. The aid is designed for patients with colorectal cancer, for whom cure is not an option. You have previously been treated with chemotherapy and now you are faced with a decision about two treatment options. Both treatment options aim to reduce your complaints as much as possible. We would like to involve you in this treatment decision.

Explaining the course of the consultation

During this consultation we will present you with information on the two different treatment options, including the adverse events and results associated with these two options. The information is intended to support your treatment decision. You can decide for yourself which information you want or do not want to receive.

This decision aid presents the chance that certain adverse events and results will occur. These chances are based on studies among large numbers of patients. It is not possible to predict whether you will actually experience these adverse events and results.

The information that is presented during the consultation is included in a take home brochure. You may want to discuss the information with others. Should you have any questions about the decision aid, please give us a call. The contact details can be found in the take home brochure.

⇒ *Do you have any questions at this moment? Please feel free to ask any questions that may arise during the consultation*

Treatment options

If you have previously been treated with chemotherapy, two different treatment options are available. The goal of both treatment options is to reduce your complaints as much as possible.

treatment option 1: best supportive care without chemotherapy

With this treatment option, your complaints will be managed using best supportive care, which may include drugs and in some cases also radiation therapy or surgery.

Best supportive care is intended to cause as few adverse events as possible. You will not receive any chemotherapy.

treatment option 2: best supportive care with chemotherapy

With this treatment option, you will not only receive best supportive care, but also chemotherapy using the drug 'irinotecan' .

As described above, your complaints will be managed using best supportive care, which may include drugs and in some cases also radiation therapy or surgery.

Alongside best supportive care, you will also receive chemotherapy in an attempt to kill cancer cells or slow down the growth of cancer cells. If successful, your complaints may be reduced. It is also possible that chemotherapy lengthens your life. On the other hand, chemotherapy can cause adverse events, which in some cases can be life threatening.

Example of information in this decision aid

The information presented in this decision aid is meant to help you choose between the two treatment options. As is often the case with decisions, there is a trade-off between pros and cons. An example of such a trade-off is presented below. The information in this example is fictitious.

When you are going on holiday, you can decide to either stay in the Netherlands, or to go abroad.

⇒ *lay down the cards for the patient according to the scheme below:*

	**holiday destination**	
	**the Netherlands**	**abroad**
**weather conditions**<br>number of people who have nice weather	**30** out of 100 do<br>**70** out of 100 don't<br>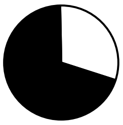	**60** out of 100 do<br>**40** out of 100 don't<br>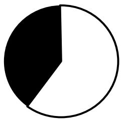
**food poisoning**<br>number of people who have food poisoning	**1** out of 100 do<br>**99** out of 100 don't<br>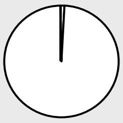	**15** out of 100 do<br>**85** out of 100 don't<br>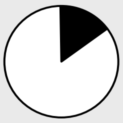

As you can see, there is a trade-off between the positive outcome (nice weather) and the negative outcome (food poisoning). Similar trade-offs can be made when it comes to treatment decisions. Throughout this decision aid, we will use the same format to present you with information on the available treatment options, to enable the trade-off between the different options.

Information on adverse events

On the next page you can find information on adverse events related to the two different treatment options.

In both treatment groups, severe and less severe adverse events can occur. Less severe events can cause discomfort, but for the sake of clarity, this decision aid will **only present severe adverse events**. Severe adverse events can lead to a hospital admission or prolongation of an admission. In rare cases, severe adverse events can be life threatening.

The length of chemotherapy treatment is on average 4 months. This decision aid shows the chance of severe adverse events occurring within this 4 month period. The aid only shows severe adverse events for which the rate of occurrence is found to be different between the two treatment options. In addition, the decision aid presents the chance that a person dies as a result of a severe adverse event.

⇒ **Do you want to receive the information on adverse events?**

If the patient decides that the information is not desired, please proceed with the next item 'response of the cancer'

Should the patient be interested in severe adverse events with an equal occurrence in both groups, these include:

constipation, fatigue, mouth ulcers, loss of appetite, skin complaints, neurological complaints, cardiovascular complaints, pain, abdominal pain, and infections.

⇒ *lay down the cards for the patient according to the scheme below:*

	**best supportive care**	
	**without chemotherapy**	**with chemotherapy**
**severe <u>nausea</u>**<br><br>you are so nauseated that your intake of food and fluid is insufficient	**3** out of 100 do<br>**97** out of 100 don't<br>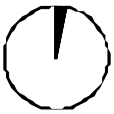	**14** out of 100 do<br>**86** out of 100 don't<br>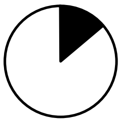
**severe <u>vomiting</u>**<br><br>you have to vomit at least 6 times a day<br>sometimes intravenous fluids and intravenous feeding are needed	**8** out of 100 do<br>**92** out of 100 don't<br>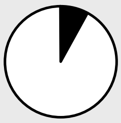	**14** out of 100 do<br>**86** out of 100 don't<br>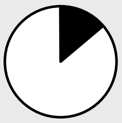
**severe <u>diarrhoea</u>**<br><br>you have an increase of at least 7 stools per day, and may also have problems controlling your bowels	**6** out of 100 do<br>**94** out of 100 don't<br>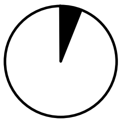	**22** out of 100 do<br>**78** out of 100 don't<br>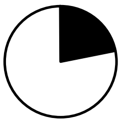
**severe <u>hair loss</u>**<br><br>you lose all your hair<br>(on your head)	**0** out of 100 do<br>**100** out of 100 don't<br>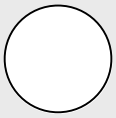	**44** out of 100 do<br>**56** out of 100 don't<br>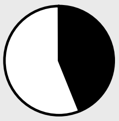
**<u>death</u>**<br><br>you die as a result of a severe adverse event	**0** out of 100 do<br>**100** out of 100 don't<br>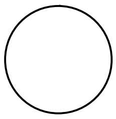	**1** out of 100 do<br>**99** out of 100 don't<br>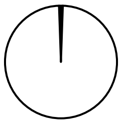

Information on the response of the cancer

The next page contains information on the response of the cancer for both treatment options.

Chemotherapy is used in an attempt to kill cancer cells or slow down the growth of cancer cells. If successful, your complaints may be reduced. During the course of chemotherapy treatment, you will have regular check-ups to monitor the response of the cancer.

There are three possible ways for the cancer to respond to treatment:

- **the size of the cancer is reduced**
  The cancer is reduced to half or less of its initial size.

- **the size of the cancer remains stable**
  There are no significant changes in the size of the cancer.

- **the size of the cancer is increased**
  The cancer does not respond to treatment and continues to grow.

Please note that any reduction or stabilization of the cancer will only be temporary. In the end, the cancer will always start to grow again.

⇒ **Do you want to receive the information on the response of the cancer?**

If the patient decides that the information is not desired, please proceed to the last item 'survival'

⇒ *lay down the cards for the patient according to the scheme below:*

	best supportive care	
	**without chemotherapy**	**with chemotherapy**
the size of the cancer is reduced *(in the pie diagram this part is displayed in white)*	**0** out of 100	**4** out of 100
the size of the cancer remains stable *(in the pie diagram this part is displayed in grey stripes)*	**6** out of 100	**52** out of 100
the size of the cancer is increased *(in the pie diagram this part is displayed in black)*	**94** out of 100	**44** out of 100
	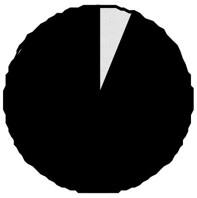	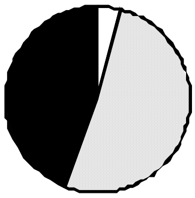

Information on survival

The next page contains information on survival for both treatment options.

In scientific studies on chemotherapy, survival is mostly reported as the time (number of months) when half the patients are expected to be alive. As an example: let's say that a 54 year old person has an expected survival of 25 years. This means that half of the people who are now 54 years old will die within the next 25 years, while the other half of the people will live beyond that time.

As a result of the cancer, people will die prematurely. This decision aid presents how many months will pass from today until half of the people are alive, for both treatment options. This can give you insight in the gain that can be expected from chemotherapy treatment.

Again, we would like to emphasize that it is not possible to predict your individual survival.

⇒ **Do you want to receive the information on survival?**

⇒ *lay down the cards for the patient according to the scheme below:*

	best supportive care	
	**without chemotherapy**	**with chemotherapy**
After this number of months,<br>50 out of 100 people are still alive and<br>50 out of 100 people will have died	**6** months	**9** months

Treatment decision

You have received information on the two different treatment options and the adverse events and results associated with these options. This information is intended to support the treatment decision about the two options. If you want to be involved in this decision, you can make a trade-off between the pros and cons of both options. An overview of all information is now laying on the table in front of you.
