# Supplementary material for: Decision aids for second-line palliative chemotherapy: a randomised phase II multicentre trial
Source: BMC Med Inform Decis Mak. 2017 Aug 31;17:130. doi: 10.1186/s12911-017-0529-y (PMC5580234; doi:10.1186/s12911-017-0529-y)
Supplement: Supplementary file 3 — Inclusion and progression form. Copy of questionnaire completed by clinicians at inclusion and progression. (PDF 79 kb) [file 12911_2017_529_MOESM3_ESM.pdf]

CLINICIAN QUESTIONNAIRE (INCLUSION/PROGRESSION)

OVERVIEW OF QUESTIONS

SECTION A: TO BE COMPLETED AT INCLUSION

Patient characteristics

Gender O male O female

Name: ………………………………………………

Address: ………………………………………………

Postcode/City: ………………………………………………

Telephone number: ………………………………………………

Date of birth: ………………………………………………

Hospital number: ………………………………………………

Name of clinician ………………………………………………

Date: …. - …. - ….

Location: ………………………………………………

Patient excluded (not asked for the study) because of:

O Insufficient Dutch language proficiency

O Labile personality structure

O Karnofsky lower than 60

O Other reason ………………………………………………………………………….........

If the patient agrees to be contacted about the study

Please complete the questions on the next page after the consultation.

If the patient does not want to be contacted about the study

Did the patient volunteer a reason for not wanting to take part? Please document below:

……………………………………………………………………………………………………

……………………………………………………………………………………………………

Questions for the clinician, to be completed after the consultation:

1. Do you think this patient will desire information about the risk of adverse events?
   O Yes
   O No

2. Do you think this patient will desire information about the chance of achieving a tumour response?
   O Yes
   O No

3. Do you think this patient will desire information about estimated survival?
   O Yes
   O No

4. Location primary tumour
   O Breast
   O Colon /rectum

SECTION B: TO BE COMPLETED AT PROGRESSION

5. Is this patient eligible for second-line palliative chemotherapy?
   O Yes
   O No --> there is no need to complete question 11

   Please explain why the patient is not eligible

   ....

   ....

   ....
