# Supplementary material for: Decision aids for second-line palliative chemotherapy: a randomised phase II multicentre trial
Source: BMC Med Inform Decis Mak. 2017 Aug 31;17:130. doi: 10.1186/s12911-017-0529-y (PMC5580234; doi:10.1186/s12911-017-0529-y)
Supplement: Supplementary file 4 — Questionnaire decision aid. Copy of nurse questionnaire. (PDF 89 kb) [file 12911_2017_529_MOESM4_ESM.pdf]

Additional File 4 - Questionnaire for consultation decision aid

To be completed during the consultation in which the decision aid is offered

---

Questions for the patient:

1. Which treatment do you prefer at this time?
   O treatment with chemotherapy
   O treatment without chemotherapy
   O don't know (please skip question 2 and proceed to offer the decision aid)

2. How strong is the above preference?

O	O	O	O
not strong	rather strong	strong	very strong

⇒ **Please offer the decision aid to the patient**

3. Did the patient want to receive the information about adverse events? O Yes O No
4. Did the patient want to receive the information about tumour response? O Yes O No
5. Did the patient want to receive the information about survival? O Yes O No

---

Questions for the patient:

6. Did you receive any information about adverse events from your doctor? O Yes O No
7. Did you receive any information about tumour response from your doctor? O Yes O No
8. Did you receive any information about survival from your doctor? O Yes O No

9. Which treatment do you prefer at this time?
   O treatment with chemotherapy
   O treatment without chemotherapy
   O don't know (please skip question 10)

10. How strong is the above preference?

O	O	O	O
not strong	rather strong	strong	very strong

End of this consultation, please complete the questions on the next page after the consultation.

Questions for the nurse:

11. Did the patient seem nervous, distressed, or emotional? O Yes O No

12. How did the interview go?

O	O	O	O	O
very difficult	difficult	neutral	well	very well

13. Was the patient alone or accompanied by a friend or relative? O alone O accompanied

14. Which chemotherapeutic regimen was discussed? ………..
