# Supplementary material for: Decision aids for second-line palliative chemotherapy: a randomised phase II multicentre trial
Source: BMC Med Inform Decis Mak. 2017 Aug 31;17:130. doi: 10.1186/s12911-017-0529-y (PMC5580234; doi:10.1186/s12911-017-0529-y)
Supplement: Supplementary file 5 — Patient questionnaire 1. Copy of patient questionnaire 1 (T1, baseline). (PDF 236 kb) [file 12911_2017_529_MOESM5_ESM.pdf]

Additional File 5 - Patient questionnaire 1: Baseline

---

1) General health

How would you describe your general health during the past week?

worst imaginable health state	0 - 1 - 2 - 3 - 4 - 5 - 6 - 7 - 8 - 9 - 10	best imaginable health state

2) Mental adjustment to cancer scale [51]

3) Adapted Lerman's Cancer Worry Scale [50]

		Not at all	Sometimes	Often	Always
1	Have you thought about cancer in the past week?	1	2	3	4
2	Have these thoughts affected your mood?	1	2	3	4
3	Have these thoughts interfered with your ability to do daily activities?	1	2	3	4

4) Subjective knowledge

The following 3 questions are about how you judge your knowledge about your current or previous cancer treatments. On a scale of 1-10, where

1= extremely poor	6= sufficient
2= very poor	7= more than sufficient
3= poor	8= good
4= strongly insufficient	9= very good
5= insufficient	10= excellent

1. How would you judge your knowledge about the advantages of cancer treatments? ....
2. How would you judge your knowledge about the disadvantages of cancer treatments? ....
3. How would you judge your knowledge about cancer? ....

6. Participation preference

When the risks and benefits of the treatment options are known to you,

		Doctor alone	Mostly the doctor	Both equally	Mostly me	Me alone
1	Who decides how acceptable those risks and benefits are for you?	1	2	3	4	5
2	Who chooses between treatment options?	1	2	3	4	5

7. Information preference

Please circle the number that best indicates your view

I want to know nothing about the illness and its treatment	0 - 1 - 2 - 3 - 4 - 5 - 6 -7 - 8 - 9 - 10	I want to know everything there is to know about the illness and its treatment

8. Amount of information

Please indicate what best indicates your view about how much information you have received about cancer and its treatment

O	O	O	O	O	O	O
way too little information	too little information	slightly too little information	the right amount of information	slightly too much information	too much information	way too much information

9. Hospital Anxiety and Depression Scale (HADS) [48]

10. Health-related quality of life: EORTC QLQ-C15-PAL [49]
