# Supplementary material for: Decision aids for second-line palliative chemotherapy: a randomised phase II multicentre trial
Source: BMC Med Inform Decis Mak. 2017 Aug 31;17:130. doi: 10.1186/s12911-017-0529-y (PMC5580234; doi:10.1186/s12911-017-0529-y)
Supplement: Supplementary file 6 — Patient questionnaire 2. Copy of patient questionnaire 2 (T2, one week follow-up). (PDF 402 kb) [file 12911_2017_529_MOESM6_ESM.pdf]

PATIENT QUESTIONNAIRE 2: 1 WEEK FOLLOW UP (T2)

OVERVIEW OF QUESTIONS

---

1) General health

How would you describe your general health during the past week?

worst imaginable health state	0 - 1 - 2 - 3 - 4 - 5 - 6 - 7 - 8 - 9 - 10	best imaginable health state

2) Treatment choice and strength of treatment preference

Which treatment do you prefer at this time?
O best supportive care **with** chemotherapy
O best supportive care **without** chemotherapy
O don't know (please skip the next question)

How strong is the above preference?

O	O	O	O
not strong	rather strong	strong	very strong

3) Amount of information

Please indicate what best indicates your view about how much information you have received about cancer treatment over the past few weeks

O	O	O	O	O	O	O
way too little information	too little information	slightly too little information	the right amount of information	slightly too much information	too much information	way too much information

Have you received any treatment-related information you would rather not have received?

O Yes
O No

4) Mental adjustment to cancer scale [51]

5) Adapted Lerman's Cancer Worry Scale [50]

		Not at all	Sometimes	Often	Always
1	Have you thought about cancer in the past week?	1	2	3	4
2	Have these thoughts affected your mood?	1	2	3	4
3	Have these thoughts interfered with your ability to do daily activities?	1	2	3	4

6) Satisfaction with quality of information

Please indicate your level of **satisfaction** with the **quality of information**...

		Dissatisfied	Slightly dissatisfied	Neutral	Slightly satisfied	Satisfied	Very much satisfied
1	About treatment-related adverse events	1	2	3	4	5	6
2	About any effects of treatment on the size of the tumour	1	2	3	4	5	6
3	About the effect of treatment on survival	1	2	3	4	5	6

7) Balanced presentation of information

There are two treatment options: best supportive care with or without chemotherapy.

Did you feel the information about this treatment choice was presented in a balanced way?

O No, clearly in favour of BSC plus chemotherapy
O No, slightly in favour of BSC plus chemotherapy
O Yes, balanced
O No, slightly in favour of BSC alone
O No, clearly in favour of BSC alone

8) Other comments about information received

If you have any other comments about the information you received about cancer treatment over the past few weeks, please write them down below:

....

....

....

9) Evaluation of information

How unpleasant did you find the information about the available treatment options?

O	O	O	O	O
Not unpleasant	Slightly unpleasant	Quite unpleasant	Unpleasant	Very unpleasant

How shocking did you find the information about the available treatment options?

O	O	O	O	O
Not shocking	Slightly shocking	Quite shocking	Shocking	Very shocking

How frightening did you find the information about the available treatment options?

O	O	O	O	O
Not frightening	Slightly frightening	Quite frightening	Frightening	Very frightening

How threatening did you find the information about **adverse events**?

O	O	O	O	O
Not threatening	Slightly threatening	Quite threatening	Threatening	Very threatening

How threatening did you find the information about **tumour response** to treatment?

O	O	O	O	O
Not threatening	Slightly threatening	Quite threatening	Threatening	Very threatening

How threatening did you find the information about **survival?**

O	O	O	O	O
Not threatening	Slightly threatening	Quite threatening	Threatening	Very threatening

10) Information received from oncologist

In your recent meeting with the oncologist, did the oncologist provide any information about adverse events?

O Yes
O No

In your recent meeting with the oncologist, did the oncologist provide any information about tumour response (any changes in the size of the tumour) to treatment?

O Yes
O No

In your recent meeting with the oncologist, did the oncologist provide any information about estimated survival?

O Yes
O No

11) Treatment subjective knowledge

The following 3 questions are about how you judge your knowledge about the two treatment options. On a scale of 1-10, where

1= extremely poor	6= sufficient
2= very poor	7= more than sufficient
3= poor	8= good
4= strongly insufficient	9= very good
5= insufficient	10= excellent

1. How would you judge your knowledge about the advantages of cancer treatments? ....
2. How would you judge your knowledge about the disadvantages of cancer treatments? ....
3. How would you judge your knowledge about cancer? ....

12) Perceived participation

		Doctor alone	Mostly the doctor	Both equally	Mostly me	Me alone
1	Who was involved in deciding if the risks and benefits of treatment were acceptable for you?	1	2	3	4	5
2	Who was involved in choosing between treatment options?	1	2	3	4	5

Do you feel you had a choice between the treatment options?
- O Yes
- O No

Do you feel your opinion mattered when choosing between treatment options?
- O Yes
- O No

13) Hospital Anxiety and Depression Scale (HADS) [48]

14) Objective risk

Please provide your best estimate of the risk of experiencing severe diarrhoea as a side effect of chemotherapy .... %

Please provide your best estimate of the chance that chemotherapy will shrink the tumour by 50% .... %

15) Objective knowledge

1	If treated with chemotherapy, no radiotherapy will be necessary	Right	Wrong
2	When the tumour is growing, chemotherapy is the only way to ease symptoms	Right	Wrong
3	The main goal of best supportive care is to ease symptoms	Right	Wrong
4	Treatment with chemotherapy will prolong survival by 6 months on average	Right	Wrong
5	Patients receiving best supportive care alone experience more pain than patients receiving chemotherapy	Right	Wrong

16) Subjective risk

In my opinion, the risk of experiencing adverse events related to chemotherapy is:

O Very high
O High
O Neither high nor low
O Low
O Very low

In my opinion, when receiving treatment with chemotherapy - compared to best supportive care alone- the risk of the tumour continuing to grow is:

O Much higher
O Higher
O Slightly higher
O Neither higher nor lower
O Slightly lower
O Lower
O Much lower

In my opinion, when receiving treatment with chemotherapy - compared to best supportive care alone- the risk of experiencing pain is:

O Much higher
O Higher
O Slightly higher
O Neither higher nor lower
O Slightly lower
O Lower
O Much lower

17) Decision Evaluation Scale [54]

18) Treatment attitudes: valuations

How appropriate do you feel treatment with **best supportive care alone** would be for you? .... (0-10)

How appropriate do you feel treatment with **chemotherapy** would be for you? .... (0-10)

19) Health-related quality of life: EORTC QLQ-C15-PAL [49]
