# Supplementary material for: Decision aids for second-line palliative chemotherapy: a randomised phase II multicentre trial
Source: BMC Med Inform Decis Mak. 2017 Aug 31;17:130. doi: 10.1186/s12911-017-0529-y (PMC5580234; doi:10.1186/s12911-017-0529-y)
Supplement: Supplementary file 7 — Patient questionnaire 3. Copy of patient questionnaire 3 (T3, eight week follow-up). (PDF 103 kb) [file 12911_2017_529_MOESM7_ESM.pdf]

PATIENT QUESTIONNAIRE 3: 8 WEEK FOLLOW UP (T3)

OVERVIEW OF QUESTIONS

---

1) General health

How would you describe your general health during the past week?

worst imaginable health state	0 - 1 - 2 - 3 - 4 - 5 - 6 - 7 - 8 - 9 - 10	best imaginable health state

2) Treatment received

Which treatment did you receive?
O best supportive care **with** chemotherapy
O best supportive care **without** chemotherapy
O don't know

3) Treatment satisfaction

Please indicate your level of **satisfaction** with...

		Dissatisfied	Slightly dissatisfied	Neutral	Slightly satisfied	Satisfied	Very much satisfied
1	The treatment received	1	2	3	4	5	6
2	The physical consequences of treatment	1	2	3	4	5	6
3	The emotional consequences of treatment	1	2	3	4	5	6

4) Mental adjustment to cancer scale [51]

5) Adapted Lerman's Cancer Worry Scale [50]

		Not at all	Sometimes	Often	Always
1	Have you thought about cancer in the past week?	1	2	3	4
2	Have these thoughts affected your mood?	1	2	3	4
3	Have these thoughts interfered with your ability to do daily activities?	1	2	3	4

6) Satisfaction with quality of information

Please indicate your level of **satisfaction** with the **quality of information**...

		Dissatisfied	Slightly dissatisfied	Neutral	Slightly satisfied	Satisfied	Very much satisfied
1	About treatment-related adverse events	1	2	3	4	5	6
2	About any effects of treatment on the size of the tumour	1	2	3	4	5	6
3	About the effect of treatment on survival	1	2	3	4	5	6

7) Amount of information

Please indicate what best indicates your view about how much information you have received about cancer treatment over the past few weeks

O	O	O	O	O	O	O
way too little information	too little information	slightly too little information	the right amount of information	slightly too much information	too much information	way too much information

8) Perceived participation

		Doctor alone	Mostly the doctor	Both equally	Mostly me	Me alone
1	Who was involved in deciding if the risks and benefits of treatment were acceptable for you?	1	2	3	4	5
2	Who was involved in choosing between treatment options?	1	2	3	4	5

Do you feel you had a choice between the treatment options?
O Yes
O No

Do you feel your opinion mattered when choosing between treatment options?
O Yes
O No

9) Hospital Anxiety and Depression Scale (HADS) [48]

10) Decision Evaluation Scale [54]

11) Treatment attitudes: valuations

On a scale of 1-10, where

1= extremely poor	6= sufficient
2= very poor	7= more than sufficient
3= poor	8= good
4= strongly insufficient	9= very good
5= insufficient	10= excellent

1. How would you value the treatment you received? ....

If you could choose again..

How appropriate do you feel treatment with **best supportive care alone** would be for you? .... (0-10)

How appropriate do you feel treatment with **chemotherapy** would be for you? .... (0-10)

12) Health-related quality of life: EORTC QLQ-C15-PAL [49]
